# Geranylgeranylacetone selectively binds to the HSP70 of *Helicobacter pylori* and alters its coccoid morphology

**DOI:** 10.1038/srep13738

**Published:** 2015-09-08

**Authors:** Ewa Grave, Shin-ichi Yokota, Soh Yamamoto, Arisa Tamura, Takako Ohtaki-Mizoguchi, Kenji Yokota, Keiji Oguma, Kazuhiko Fujiwara, Nobuaki Ogawa, Tomoya Okamoto, Michiro Otaka, Hideaki Itoh

**Affiliations:** 1 Department of Life Science, Graduate School and Faculty of Engineering Science, Akita University, Akita 010-8502, Japan; 2 Department of Microbiology, Sapporo Medical University School of Medicine, Sapporo, 060-8556, Japan; 3 Graduate School of Health Sciences, Okayama University, Okayama 700-8558, Japan; 4 Department of Bacteriology, Okayama University Graduate School of Medicine, Dentistry and Pharmaceutical Sciences, Okayama 700-8558, Japan; 5 Department of Gastroenterology, Juntendo University School of Medicine, Bunkyo-Ku, Tokyo 113-8421, Japan

## Abstract

Geranylgeranylacetone (GGA) is used to treat patients suffering from peptic ulcers and gastritis. We examined the effect of GGA on *Helicobacter pylori*, which is a causative factor of gastrointestinal diseases. Previously, we have reported that GGA binds specifically to the molecular chaperone HSP70. In this paper, we report that GGA bounds to *H. pylori* HSP70 (product of the DnaK gene) with 26-times higher affinity than to human HSP70, and induced large conformational changes as observed from surface plasmon resonance and circular dichroism. Binding of GGA suppressed the activity of the *H. pylori* chaperone. GGA also altered several characteristics of *H. pylori* cells. GGA-treated cells elicited enhanced interleukin-8 production by gastric cancer cell lines and potentiated susceptibility to complement as compared to untreated cells. GGA also caused morphological alterations in *H. pylori* as reflected in fewer coccoid-like cells, suggesting that GGA converts *H. pylori* to an actively dividing, spiral state (vegetative form) from a non-growing, coccoid state. This morphological conversion by GGA resulted in accelerated growth of *H. pylori*. These results suggest a model in which GGA sensitizes *H. pylori* to antibiotic treatment by converting the cells to an actively growing state.

The microaerophilic gram-negative bacterium *Helicobacter pylori* can colonize the human stomach. It survives in the stomach by neutralizing gastric acid with ammonium ion, which is produced from urea by the action of *H. pylori* urease. The bacterium adheres on gastric epithelial cells via adhesions, and grows in the mucus layer. *H. pylori* is an important etiological agent of gastroduodenal diseases, such as gastritis, gastric and duodenal ulcers, gastric cancer, and mucosa-associated lymphoid tissue (MALT) lymphoma^1^. During prolonged chronic infection, the major cause of the symptoms is the resultant inflammatory reaction^2^,^3^. *H. pylori*-induced diseases are primarily treated with eradication therapy by antibiotics. The first-line eradication regimen is a three-drug combination therapy using a proton pump inhibitor (such as lansoprazole, omeprazole, rabeprazole, or esomeprazole), amoxicillin, and clarithromycin. Recently, failures of this treatment have increased, because of the appearance of antibiotic-resistant (especially clarithromycin) *H. pylori*. Metronidazole and sitafloxacin, instead of clarithromycin, are recommended for second and third line therapies in Japan^4^.

*H. pylori* cells in the biopsy specimens from patient stomachs showed elongated rod-shaped or coccoid morphologies in variable proportions^5^,^6^. The predominance of rods in exponentially growing *in vitro* cultures suggests that this form represents proliferating cells, namely the vegetative form. On the other hand, the coccoid form is recognized as a viable-but-not-culturable state, which is resistant to various environmental stresses, such as starvation. The coccoid form could contaminate environments such as drinking water, thereby leading to oral transmission.

Geranylgeranylacetone (6,10,14,18-tetramethyl-5,9,13,17-nonadecatetraen-2-one; GGA) is an anti-peptic ulcer drug developed and approved in Japan in 1984. GGA is shown to suppress *H. pylori*-induced tissue and cell injury^7^,^8^ and inflammatory reaction^9^,^10^, so it is expected to show beneficial effects on *H. pylori*-infected tissues. One of pharmacological actions of GGA is induction of molecular chaperone 70-kDa heat shock protein (HSP70) in mammals^11^. The induced HSP70 protects the gastrointestinal tract from cell damage induced by various stresses, such as reactive oxygen, ethanol and non-steroidal anti-inflammatory drugs (NSAIDs)^11^,^12^. In addition, mammalian HSP70 induced by GGA has been reported to be cytoprotective in a number of pathological lesions other than the gastrointestinal tract, including heart ischemia^13^, hepatectomy^14^, cerebral infarction^15^, and colitis^16^.

The ubiquitous HSP70s and their constitutive cognates (HSC70s) are highly conserved in evolution. HSP70s are present in mammalian cells as two different gene products that are closely related: a stress-inducible form, HSP70 (known as HSP72), and a constitutively expressed form, HSC70 (known as HSP73)^17^. HSP70s consist of two domains, a highly conserved NH2-terminal ATPase domain with a molecular mass of 45 kDa and a COOH terminal domain of 25 kDa^18^,^19^. The polypeptide-binding site is located in the 18-kDa of the COOH-terminal adjacent to the ATPase domain^20^,^21^. The ATP-bound form of HSP70 binds and releases peptide rapidly, whereas the ADP form binds peptide slowly and in a more stable complex^22^,^23^. The C-terminal peptide-binding domain of the HSC70 binds to heat shock factor-1 (HSF-1) and the activation of HSF-1 is suppressed under normal conditions. We have previously reported that HSC70 and HSP70 specifically bind to a GGA-affinity matrix^24^. When concentration of the GGA is increased in the cell, GGA preferentially binds to the C-terminal of HSC70 and dissociates HSF-1. The released HSF-1 is phosphorylated and activated, acquiring the ability to bind to a heat shock element (HSE) in the promoter of the heat-inducible HSP70 gene and resulting in induction of HSP70 expression.

HSPs are highly conserved between mammals and bacteria, so we anticipated that GGA could also bind to the HSP70 of *H. pylori* (product of the DnaK gene). It is important to establish whether GGA acts on both human and *H .pylori* cells. A previous report showed that GGA has antibacterial activity against *H. pylori*, but not *Campylobacter jejuni*^25^. In the present study, we investigated the properties of binding of GGA to both *H. pylori* and human HSP70, and also the effect of GGA on *H. pylori* cells.

## Results

### Purification of human and *H. pylori* HSP70s

**Figure 1:**
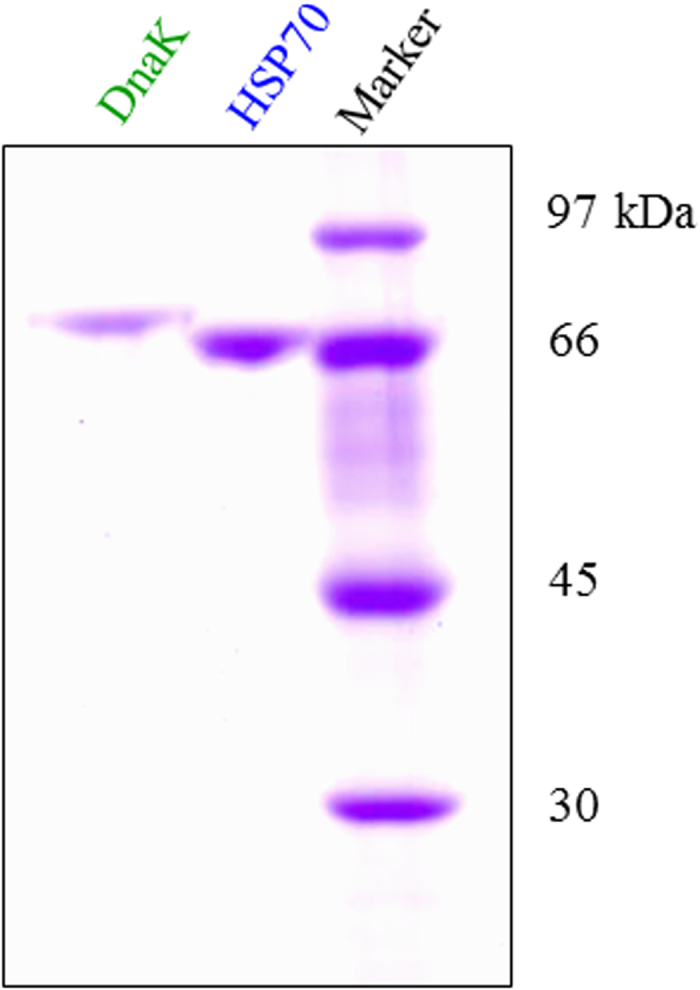
Purified DnaK and HSP70. DnaK and HSP70 were purified as described under “Materials and Methods” and the purity was analyzed by SDS-PAGE (9% gels).

We investigated the sequence homology between human HSP70 and *H. pylori* DnaK. It has been shown that HSP70 is composed of two domains, ATPase and peptide-binding domain. The ATPase and peptide-binding domains are located in the N-terminal and C-terminal halves of the protein, respectively. There was 47% sequence identity between those two proteins, which was evenly distributed across the two halves. There are some differences in the peptide-binding domains (Supplementary Figure S1). After expression and purification, the human and *H. pylori* proteins were compared by SDS-PAGE (Fig. 1). The apparent molecular weight of the *H. pylori* protein (referred to henceforth as DnaK) was slightly larger. These purified proteins were used in the present study.

### Affinity of GGA for HSP70 and DnaK

**Figure 2:**
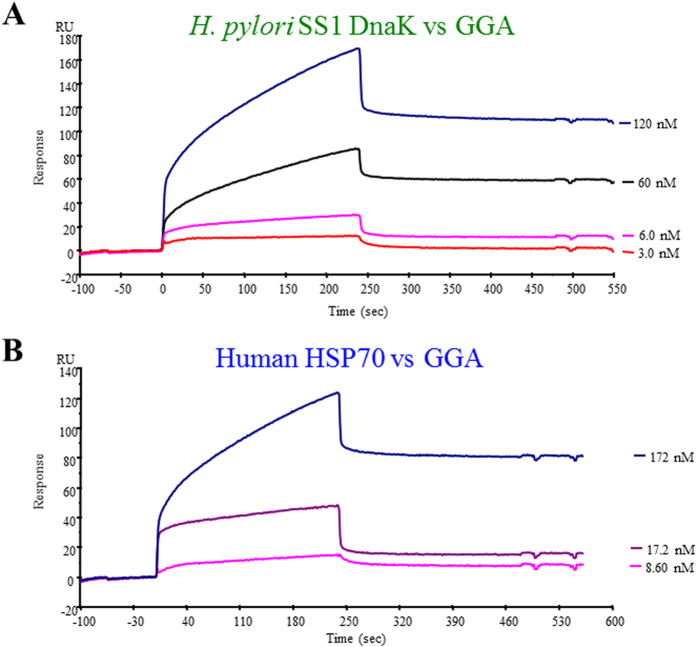
Surface plasmon resonance analysis of the interaction between HSP70 or DnaK and GGA. A sensorgram for the binding of DnaK (**A**) or HSP70 (**B**) to GGA is shown. Different concentrations of GGA were injected as described under “Materials and Methods”. RU, resonance units.

A sensorgram from surface plasmon resonance of the binding of GGA to HSP70 and DnaK is shown in Fig. 2. The kinetic parameters K_a_, K_d_, and K_D_ were estimated using BIAcore evaluation software, fitting the binding curves to a simple bimolecular binding algorithm. Based on these results with immobilized HSP70 and soluble GGA, the apparent equilibrium dissociation constant (K_D_) was 2.00 × 10^−6^ M. In contrast, the K_D_ value of DnaK and GGA was calculated as 7.55 × 10^−8^ M. Thus, the affinity of GGA to the bacterial DnaK is about 26 times higher than that to mammalian HSP70.

### Changes in HSP70 and DnaK conformation upon binding of GGA

**Figure 3:**
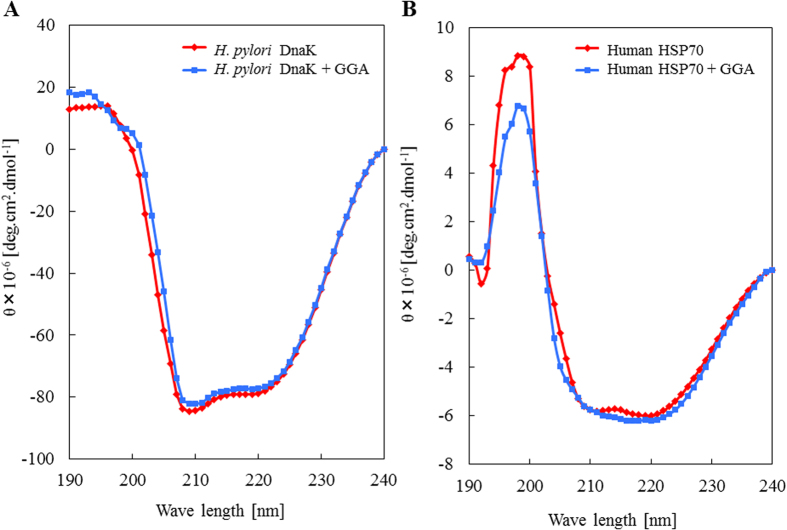
Effect of GGA on the conformational changes of DnaK and HSP70. (**A**) the CD spectrum of DnaK was measured in the absence (closed red diamond) or presence (closed blue square) of GGA as described under “Materials and Methods” denotes the mean residue ellipticity. (**B**) the CD spectrum of HSP70 was measured in the absence (closed red diamond) or presence (closed blue square) of GGA as described under “Materials and Methods” the mean residue ellipticity.

We investigated whether GGA may give rise to conformational change in HSP70 and DnaK using far UV Circular Dichroism (CD) spectra. GGA induced changes in DnaK spectrum (Fig. 3A) that were consistent with decreased β-sheet and increased α-helix, β-turn, and random structure [α-helix (22.0 ± 1.4 to 24.0 ± 1.0), β-sheet (16.2 ± 2.3 to 9.4 ± 3.0* (*p < 0.05), β-turn (25.2 ± 1.4 to 28.3 ± 1.6), and random structure (36.5 ± 0.9 to 38.3 ± 0.8)]. In contrast, GGA caused only slight conformational changes in HSP70 (Fig. 3B) [α-helix (26.4 ± 3.9 to 27.0 ± 6.4), β-sheet (27.0 ± 0.7 to 24.4 ± 2.9), β-turn (26.1 ± 2.5 to 26.1 ± 5.2), and random structure (20.4 ± 1.7 to 22.4 ± 4.6)]. The conformational changes in HSP70 and DnaK in the presence or absence of GGA are summarized in Table 1.

### Effect of GGA on chaperone activity of DnaK and HSC70

**Figure 4:**
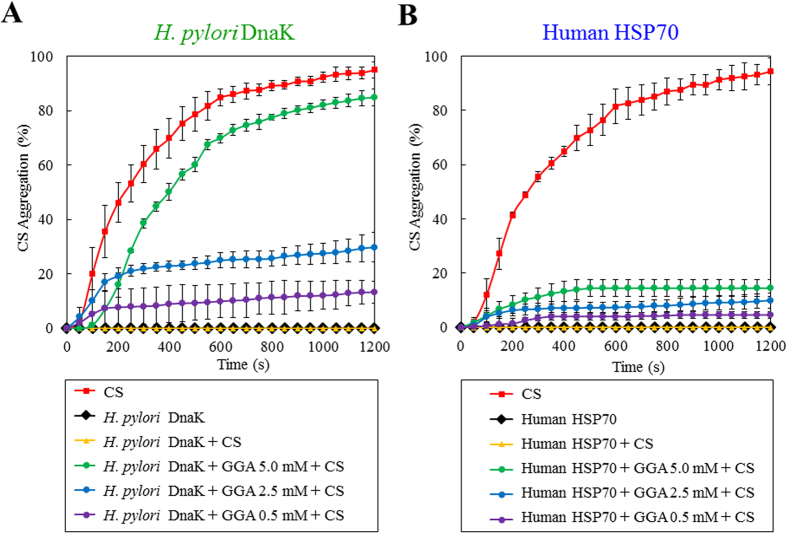
Effect of GGA on the chaperone activity of DnaK and HSP70. (**A**) time course of 50 μM DnaK (closed black diamond), 0.25 μM citrate synthase (CS) aggregation in buffer (closed red square), buffer containing 50 μM DnaK (closed yellow triangle), buffer containing 50 μM DnaK and 0.5 mM GGA (closed purple circle), buffer containing 50 μM DnaK and 2.5 mM GGA (closed blue circle), and buffer containing 50 μM DnaK and 5.0 mM GGA (closed green circle). (**B)** time course of 50 μM HSP70 (closed black diamond), 0.5 M citrate synthase aggregation in buffer (closed red square), buffer containing 50 μM HSP70 (closed yellow triangle), buffer containing 50 μM HSP70 and 0.5 mM GGA (closed purple circle), buffer containing 50 μM HSP70 and 2.5 mM GGA (closed blue circle), and buffer containing 50 μM HSP70 and 5.0 mM GGA (closed green circle).

We compared the effect of GGA on the chaperone activities of DnaK and HSP70. Citrate synthase (CS) is very unstable to heat (see Fig. 4 for the thermal aggregation assay). Both DnaK (Fig. 4A) and HSP70 (Fig. 4B) inhibited CS aggregation completely. GGA had only slight effect on chaperone activity of HSP70 (Fig. 4B). HSP70 activity was reduced by only 15% at 5 mM GGA; and reduced by 10 and 5% 2.5 and 0.5 mM, respectively. In contrast, the chaperone activity of DnaK was inhibited almost completely at GGA 5 mM (Fig. 4A). These results are consistent with the influence of the drug on the conformations of the two proteins.

### Effect of GGA on growth and morphology of *H. pylori*

**Figure 5:**
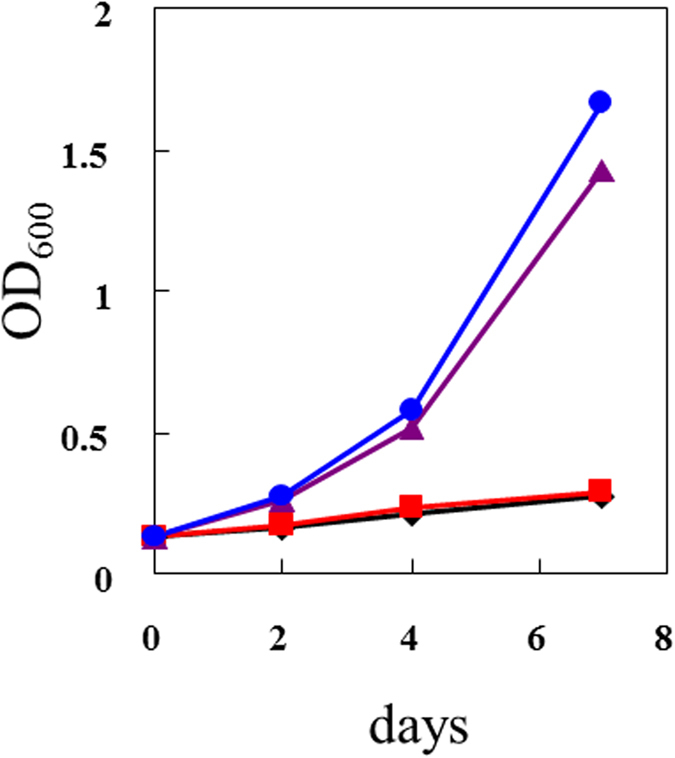
Effect of GGA on *H. pylori* cell growth. GGA was added to *H. pylori* SS1 culture in BHI-FBS broth at 37 ^o^C under microaerophilic condition at a concentration of 0 (closed black diamond), 0.2 (closed red square), 1 (closed purple triangle), or 5 (closed blue circle) mM. The cells were cultured, and turbidity (determined by A_600_) was measured.

**Figure 6:**
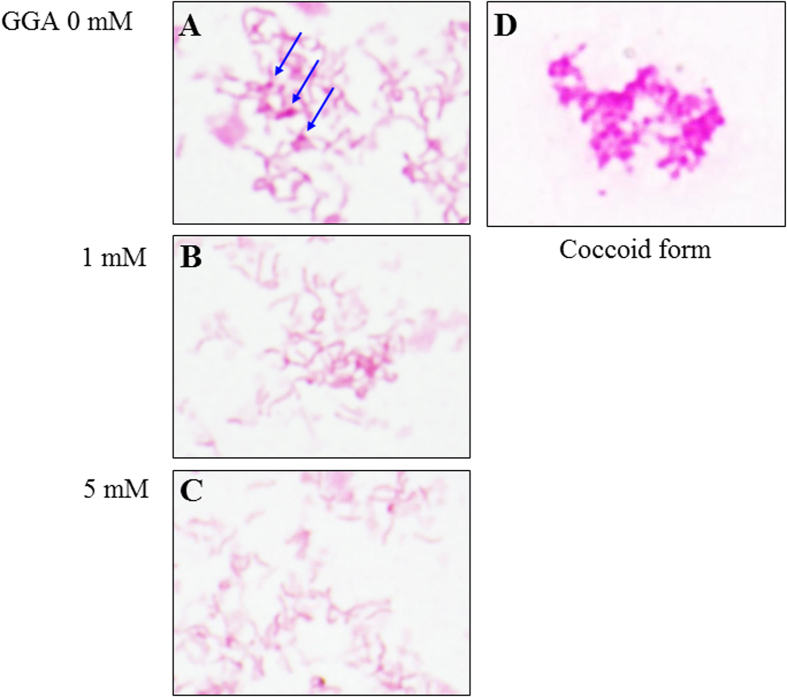
Effect of GGA on *H. pylori* cell morphology. *H. pylori* SS1 was cultured in BHI-FBS broth at 37 ^o^C under microaerophilic condition in the absence or presence of GGA at a concentration of 0 (**A**), 1 (**B**), and 5 mM (**C**). Bacterial smear was stained by Gram staining. Coccoid form cells (**D**) were prepared by cultivation under anaerobic condition. Arrows in (**A**) indicate coccoid-like cells.

In broth culture medium, GGA accelerated the growth of *H. pylori* in a dose-dependent manner (Fig. 5). Microscopic observation of the cells from these cultures revealed a mixture of Gram-negative rods and small spherical forms. A Gram stained micrograph of typical coccoid-form cells was shown in Fig. 6-D as a control. The coccoid-form cells were round and deeper pink than the rod-shaped cells. GGA caused a disappearance of coccoid-like form cells in a dose-dependent manner (Fig. 6). Therefore, GGA decreased the occurrence of the viable-but-not-culturable coccoid form, and increased highly-dividing rod-shaped cells in these cultures of *H. pylori*.

### Effect of GGA on serum sensitivity of *H. pylori*

**Figure 7:**
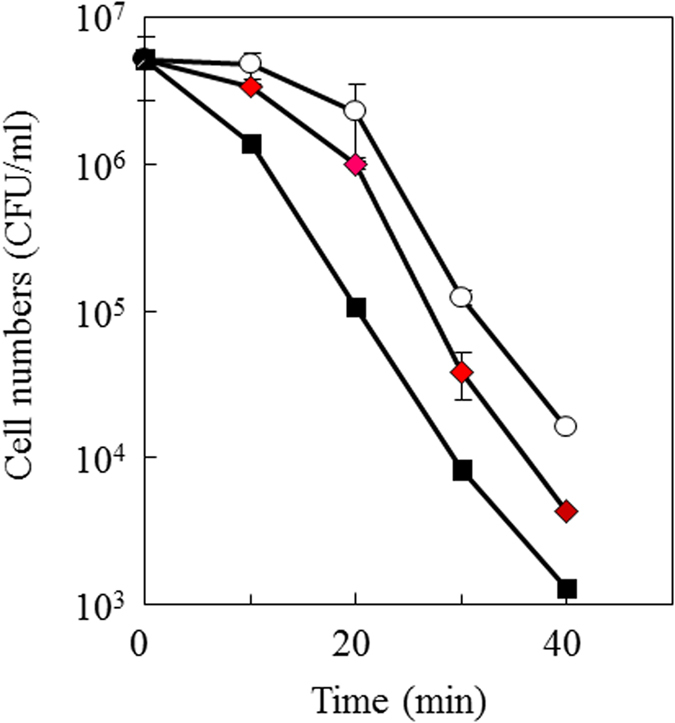
Effect of GGA on serum sensitivity of *H. pylori* cells. *H. pylori* SS1 was precultured in the absence or presence of GGA at a concentration of 0 (open circle), 1 (closed red diamond), and 5 (closed black square) mM. The resulting cells were treated with 50% normal rabbit serum as a source of complement. After incubation at various times, the suspension was diluted appropriately and plated on sheep blood agar plates. After 72 h culture, the colonies were counted. Each experiment was performed in quadruplicate.

The changes of cell morphology suggest that cell surface of *H. pylori* may be altered by GGA treatment. So we examined effect of GGA treatment on serum sensitivity, namely antibody-independent complement-mediated killing, by using normal rabbit serum as a source of complement. GGA-treated *H. pylori* cells were more rapidly killed by the serum than untreated cells (Fig. 7), consistent with the GGA-treated cells being more susceptible to complement.

### Effect of GGA on induction of interleukin-8 (IL-8) in gastric cancer cell lines by *H. pylori*

**Figure 8:**
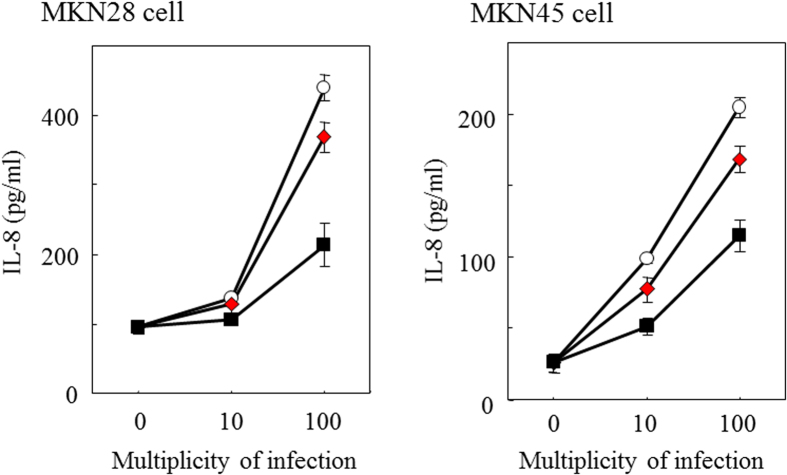
Effect of GGA on Interleukin-8 (IL-8) production induced by *H. pylori* live cells in gastric carcinoma cell line MKN28 and MKN45. *H. pylori* SS1 was precultured in the absence or presence of GGA at a concentration of 0 (open circle), 1 (closed red diamond), and 5 (closed black square) mM. The resulting cells were inoculated to MKN28 cells or MKN45 cells at MOI 10 or 100. After 24 h incubation, IL-8 in the culture supernatant was measured by ELISA. Each experiment was performed in triplicate.

We examined effect of GGA pretreatment of *H. pylori* cells on their induction of IL-8 in gastric carcinoma cell lines, MKN28 and MKN45. IL-8 is the most important chemokine produced by epithelial cells for the inflammatory response induction and pathogenesis of *H. pylori*^26^. GGA pretreatment decreased the ability of *H. pylori* to induce IL-8 in both cancer cell lines (Fig. 8).

## Discussion

GGA is a gastromucoprotective drug with a low incidence of side effects. GGA is employed to treat gastritis/gastric ulcers in Japan and other areas of Asia. GGA is commonly employed as an HSP inducing agent. HSP promotes protein folding/membrane passage and protects cells against various types of stressors including infection and inflammation. Organs/cells in which HSP has been induced show a potent activity to resist toxic substances^11^. There are many reports of *in vivo* experiments, including a human clinical study^27^, that GGA induces HSPs responsible for cell protection, for example, inhibiting NSAID-related acute gastric/small intestinal mucosal injury.

We have previously reported that mammalian HSP70/HSC70 was a GGA-specific binding protein and proposed induction mechanisms of HSP70 by GGA^24^. HSPs are highly conserved protein among organisms, including prokaryote and eukaryote. The sequence homology between human HSP70 and *H. pylori* DnaK is 47%. So we anticipated GGA binding to *H. pylori* DnaK. In this study, we investigated binding of GGA to *H. pylori* DnaK and the influence of GGA on *H. pylori* viable cells. GGA bound to *H. pylori* DnaK with higher affinity than to human HSP70. The same concentration of GGA selectively affected the conformation and chaperone activity of *H. pylori* DnaK compared to those of human HSP70. There are some differences in amino acid sequences of the peptide binding domain of human HSP70 and *H. pylori* DnaK. The sequence differences could result in different affinities for GGA.

GGA shares cell protective effects, especially gastric mucosa cells. There are a few reports that GGA protects from *H. pylori*-induced gastric injury. The reduction of *H. pylori*-induced gastric mucosa injury by GGA was shown by using a rat *in vivo* mode^17^. GGA attenuates growth suppression of human umbilical vein epithelial cells induced by *H. pylori* cell extract, and the authors speculate that GGA could recover host angiogenesis, which is important for the healing of gastric ulcer^8^. So GGA is a promising agent that is effective against *H. pylori*-induced pathologies. Effects of GGA on *H. pylori* cells and *H. pylori*-induced immune responses have been found in several reports and were observed in our present study. Ishii reported that GGA showed antibacterial activity to *H. pylori*, however, it highly depended on supplements to media^25^. The antibacterial activity was observed on Brucella agar containing albumin, charcoal, or egg yolk emulsion, but it was not observed on the agar containing horse defibrinated blood or β-cyclodextrin. Conversely, our present study indicated that GGA accelerated growth rate of *H. pylori* in BHI-FBS broth (Fig. 5) and Brucella broth containing FBS (data not shown). An explanation of the growth enhancement was that GGA converted *H. pylori* to vegetative phase from the coccoid form (Fig. 6). The coccoid form of *H. pylori* is recognized as a viable-but-not-culturable state that is more resistant to environmental stresses than the vegetative state with active proliferation. Treatment with antibiotics, including β-lactams, such as amoxicillin, causes conversion of *H. pylori* to the coccoid form^28^,^29^,^30^. Generally, antibiotics are effective on actively proliferating cells and, in fact, the coccoid form of *H. pylori* is less susceptible to antibiotic treatment^30^,^31^. This incomplete eradication could explain relapse of *H. pylori*-induced gastric disorder after the antibiotic treatment^32^. GGA is expected promote eradication of *H. pylori* through conversion to the vegetative form.

*H. pylori* is susceptible to antibody-independent complement killing^33^. We also observed this serum sensitivity of *H. pylori* and, furthermore, GGA-treated cells were more susceptible. It seems that the morphological change from the coccoid to vegetative form reflects an alternation of cell surface properties, resulting in greater sensitivity to complement killing.

Effects of GGA on a *H. pylori* cell-induced inflammatory response, IL-8 production, have been reported^9^,^10^. In these reports, live *H. pylori* cells and GGA were simultaneously incubated with human gastric carcinoma cell lines, such as KATOIII and MKN28. It is unclear if GGA affected the gastric or *H. pylori* cells. We performed this experiment under conditions in which *H. pylori* cells were pre-treated with GGA and then cocultured with human gastric cells under GGA-free conditions. We therefore suggest that suppression of IL-8 induction occurred by direct action of GGA on the bacteria. Induction of NF-κB activation and proinflammatory cytokine production has been shown to be mediated by the type IV secretion system of bacterial cells^34^,^35^. The type IV secretion system consists of proteins encoded by genes located on cag pathogenicity islands^36^,^37^. GGA could suppress expression or function of this system. Poursina *et al.* reported that *cagE* mRNA, which encodes a key factor of type IV secretion system CagE, was expressed in coccoid cells, however, the expression levels in coccoid cells were lower than in the spiral form cells^38^. This issue needs to be clarified by future work.

In conclusion, GGA acts on both human cells and *H. pylori* cells for protection from gastric disorder. For human cells, HSP70 induced by GGA protects the gastric cells from injury induced by *H. pylori*. For the *H. pylori* cells, GGA causes inhibition of DnaK function, increase of complement susceptibility, suppression of IL-8 induction, and conversion to vegetative form from coccoid form. The sum of these actions on *H. pylori* should have a positive effect on the symptoms caused by the *H. pylori* infection. Of greatest interest, the conversion to the vegetative form should increase the susceptibility of *H. pylori* to many antibiotics.

## Materials and Methods

GGA was obtained from Eisai Co., Ltd. (Tokyo, Japan). Human HSP70 cDNA (kindly provided from Dr. Richard Morimoto, Northwestern University) was used in this study. Human HSP70 cDNA was amplified by PCR (iCycler BioRad, CA, USA) using HSP70-specific primer containing the restriction enzyme site which forward primer is encoding Xho I site 5′-CTCGAGATGGCCAAAGCCGCGGCAGTC-3′ and reverse primer encoding Xba I site 5′-TCTAGACTAATCTACCTCTCAATGGT-3′ from human HSP70 cDNA. The product obtained by PCR were inserted into Xho I/Xba I sites of the pCold I vector (TaKaRa Bio, Japan). The pCold I- human HSP70 constructs were confirmed by DNA sequencing (PRISM 3100, ABI, CA, USA).

*H. pylori* genomic DNA derived from ATCC43504 was used in this study. DnaK cDNA was amplified by PCR (iCycler BioRad, CA, USA) using DnaK-specific primer containing the restriction enzyme site which forward primer is encoding Xho I site 5′-CTCGAGATGGGAAAAGTTATTGGAATTGAT-3′ and reverse primer encoding Xba I site 5′-TCTAGAGATTAAAACCGCTCGCTTGA-3′ from *H.pylori* genomic DNA. The product obtained by PCR were inserted into Xho I/Xba I sites of the pCold I vector (TaKaRa Bio, Japan). The pCold I- *H. pylori* DnaK constructs were confirmed by DNA sequencing (PRISM 3100, ABI).

The pCold I- *H. pylori* DnaK was expressed in *Eschericha coli* BL21 (Promega, Madison, USA). The cells were grown in the LB BROTH medium (Invitrogen, CA, USA) containing 100 μg/mL ampicillin until the OD_600_ reached 0.4 ~ 0.5 at 37 ^o^C. The culture medium was left standing for 30 min to 15 ^o^C. The cells were added to 1 mM IPTG (Nacalai Tesque, Kyoto, Japan) to induce protein of interest, and incubated 24 hours at 15 ^o^C. The cells were sonicated, and centrifuged at 20,000 × g for 15 min at 4 ^o^C and the supernatants were collected. The supernatants were mixed in equal amount of Ni column apply buffer (40 mM imidazole in 10 mM Tris-HCl, pH 7.4), and applied onto Ni-NTA affinity column (GE Healthcare, Buckinghamshire, UK) equilibrated with Ni column equilibration buffer (20 mM imidazole, 300 mM NaCl in10 mM Tris-HCl, pH 7.4). After washing with Ni column wash buffer (50 mM imidazole, 300 mM NaCl in10mM Tris-HCl, pH 7.4), proteins were eluted with a linear gradient of 0.1–0.5 M imidazole in 0.3 M NaCl, 10 mM Tris-HCl, pH 7.4. *H. pylori* DnaK fractions were concentrated by ultrafiltration.

### Surface plasmon resonance (SPR) assay

Surface plasmon resonance (SPR) assay were performed as previously described^39^. All SPR measurements were performed on a BIAcore 2000 instrument (GE Healthcare, Buckinghamshire, UK) at 25 ^o^C, and 25 mM HEPES-KOH (pH 7.4) buffer containing 0.005% Tween 20, 5 mM MgCl_2_, and 150 mM KCl was used as the running buffer at the flow rate of 10 μl/min. HSP70 or DnaK (1 μM) were dissolved in 100 mM sodium acetate buffer (pH 4.0) and immobilized on CM5 sensor chips (GE Healthcare, Buckinghamshire, UK) by amino coupling. HSP70 (~15,000 RU) was immobilized on the chip, and GGA solutions (8.6, 17.2 and 172 nM) were loaded on the chip using running buffer. DnaK (~15,000 RU) was immobilized on the chip, and GGA solutions (3, 6, 60, and 120  nM) were loaded on the chip using running buffer. Regeneration of the sensor chip surface was achieved by 1-min pulse (10 μl) of 0.5 M NaCl in running buffer. The dissociation constant (K_D_) was obtained by non-linear curve fitting based on a steady-state affinity using BIAevaluation 3.0 software.

### Far-UV Circular Dichroism (CD)

The CD measurements were performed by a J-720 spectropolarimeter (Jasco, Tokyo, Japan) as previously described^40^. The CD spectrum (190–240 nm) of HSP70 (1.1 μM) or DnaK (1.1 μM) in 50 mM HEPES-NaOH buffer (pH 7.4) in the presence or absence of GGA (11.0 μM) were recorded at 25 ^o^C using a cuvette with a 0.5-mm path length. The observed specific ellipticity after normalization against a blank was converted to the mean residue ellipticity [θ] (degrees cm^2^ dmol^−1^). The secondary structure of HSP70 or DnaK was calculated using an analytical program for secondary structure of proteins (SSE-338W, Jasco, Tokyo, Japan).

### Measurement of protein aggregation and chaperone function of HSPs

The thermal aggregation of citrate synthase (CS) (Roche Diagnostics, Mannheim, Germany) was monitored at 50 ^o^C as previously described^24^. Briefly, the concentration of CS used was 0.25 μM in 50 mM Hepes buffer, pH 7.4, in the presence or absence of the HSP70 (50 μM) or DnaK (50 μM), and in the presence of each HSP70 and GGA (0.5 to 5 mM). Light scattering CS was monitored for 20 min at an optical wavelength of 500 nm by Ultrospec 3000 UV-vis spectrophotometer (GE Healthcare) equipped with a temperature control unit using semi-micro-cuvettes (0.5 ml) with a path-length of 10 mm.

### Bacterial cells and cultivation

*H. pylori* strain SS1 was donated from Dr. Adrian Lee (The University of New South Wales, Sydney, Australia). SS1 was cultured in brain heat infusion broth containing 5% (vol/vol) heat-inactivated fetal bovine serum (BHI-FBS) at 37 ^o^C in 5% CO_2_, or Helicobacter agar plate (Nissui Pharmaceuticals, Tokyo, Japan) at 37 ^o^C under microareophic condition using AnaeroPack-MicroAero (Mitsubishi Gas Chemical, Tokyo, Japan). Cell growth was determined by turbidity (measured by absorbance at 600 nm). Cell numbers were determined by colony formation using tryptic soy agar plate containing 5% (v/v) sheep blood. Coccoid form cells were prepared according to Yamaguchi *et al.*^41^. Briefly, cells on Helicobacter agar plates cultured in microaerophic condition were subsequently cultured at 37 ^o^C for 7 d under anaerobic condition using AnaeroPack-Anaero.

### Gram staining

Gram staining was performed by Hucker’s modified method using Gram Solutions (Wako Pure Chemical, Osaka, Japan).

### Serum sensitivity assays

Cultured *H. pylori* SS1 cells were adjusted at a cell density of 10^7^ cells/ml. GGA was added to the cell suspension at a concentration of 0, 1, or 5 mM, and then incubated for 48 h. The cells were collected by centrifugation, and then suspended with PBS at a cell density of approximately 10^7^ cells/ml. The aliquots (200 μl) of the cell suspension were mixed with 200 μl of pooled normal rabbit serum (Cedarlane, Ontario, Canada), and then incubated for various times. After incubation, the treated cell suspension was immediately diluted with PBS at 10 to 10,000-fold and inoculated to tryptic soy agar containing 5% sheep blood. The plates were incubated for 72 h, and the resulting colonies were counted.

### IL-8 inducing activity of *H. pylori* cells

The human gastric carcinoma cell lines MKN28 and MKN45 were obtained from the Japanese Collection of Research Biosources (JCRB; Ibaraki, Japan). MKN28 and MKN45 were routinely cultured in Dulbecco’s modified minimum essential medium supplemented with 10% (vol/vol) heat-inactivated fetal bovine serum (DMEM-FBS). *H. pylori* cells were precultured in BHI-FBS for 72 h, and then added GGA at a concentration of 0, 1, or 5 mM and further cultured for 48 h. The cells were collected by centrifugation and then washed with PBS twice. The treated cells were suspended with DMEM-FBS, and inoculated to MKN28 or MKN45 cells with approximately 70% confluency at a multiplicity of infection 10 or 100. After 24 h incubation, the amounts of IL-8 in the culture supernatants were determined by enzyme-linked immunosorbent assay (ELISA) with an ELISA Development kit for human IL-8 (R&D Systems, Minneapolis, MN).

## Additional Information

**How to cite this article**: Grave, E. *et al.* Geranylgeranylacetone selectively binds to the HSP70 of *Helicobacter pylori* and alters its coccoid morphology. *Sci. Rep.* **5**, 13738; doi: 10.1038/srep13738 (2015).

## Supplementary Material

Supplementary Information

## Figures and Tables

**Table 1:** Conformational changes in HSP70 and DnaKin the presence or absence of GGA.

(%)	HSP70 − GGA	HSP70 + GGA	DnaK − GGA	DnaK + GGA
α-helix	26.4 ± 3.9	27.0 ± 6.4	22.0 ± 1.4	24.0 ± 1.0
β-sheet	27.0 ± 0.7	24.4 ± 2.9	16.2 ± 2.3	9.4 ± 3.0*
β-turn	26.1 ± 2.5	26.1 ± 5.2	25.2 ± 1.4	28.3 ± 1.6
Random	20.4 ± 1.7	22.4 ± 4.6	36.5 ± 0.9	38.3 ± 0.8

*p < 0.05.
