# Supplementary material for: Geranylgeranylacetone selectively binds to the HSP70 of Helicobacter pylori and alters its coccoid morphology
Source: Sci Rep. 2015 Sep 8;5:13738. doi: 10.1038/srep13738 (PMC4561889; doi:10.1038/srep13738)

Geranylgeranylacetone selectively binds to the HSP70 of *H. pylori* and alters its coccoid morphology

Ewa Grave[1], Shin-ichi Yokota[2], Soh Yamamoto[2], Arisa Tamura[1], Takako Ohtaki-Mizoguchi[1], Kenji Yokota[3], Keiji Oguma[4], Kazuhiko Fujiwara[1], Nobuaki Ogawa[1], Tomoya Okamoto[1], Michiro Otaka[5], and Hideaki Itoh[1]

[1]Department of Life Science, Graduate School and Faculty of Engineering Science, Akita University, Akita 010-8502, Japan
[2]Department of Microbiology, Sapporo Medical University School of Medicine, Sapporo, 060-8556, Japan
[3]Graduate School of Health Sciences, Okayama University, Okayama 700-8558, Japan
[4]Department of Bacteriology, Okayama University Graduate School of Medicine, Dentistry and Pharmaceutical Sciences, Okayama 700-8558, Japan
[5]Department of Gastroenterology, Juntendo University School of Medicine, Bunkyo-Ku, Tokyo 113-8421, Japan

*To whom correspondence should be addressed: Hideaki Itoh.
Department of Life Science, Graduate School and Faculty of Engineering Science, Akita University, 1-1 Tegata Gakuen Town, Akita University, Akita 010-8502, Japan.
Tel and Fax: +81-18-889-3041
E-mail: itohh@ipc.akita-u.ac.jp

Supplementary Figure S1. Sequence homology between human HSP70 and *H. Pylori* SS1 DnaK (HSP70 homologue). There was 47% homology between HSP70 and DnaK. The ATPase- and peptide binding domain of homology was 48- and 45%, respectively.

Supplementary Figure S1

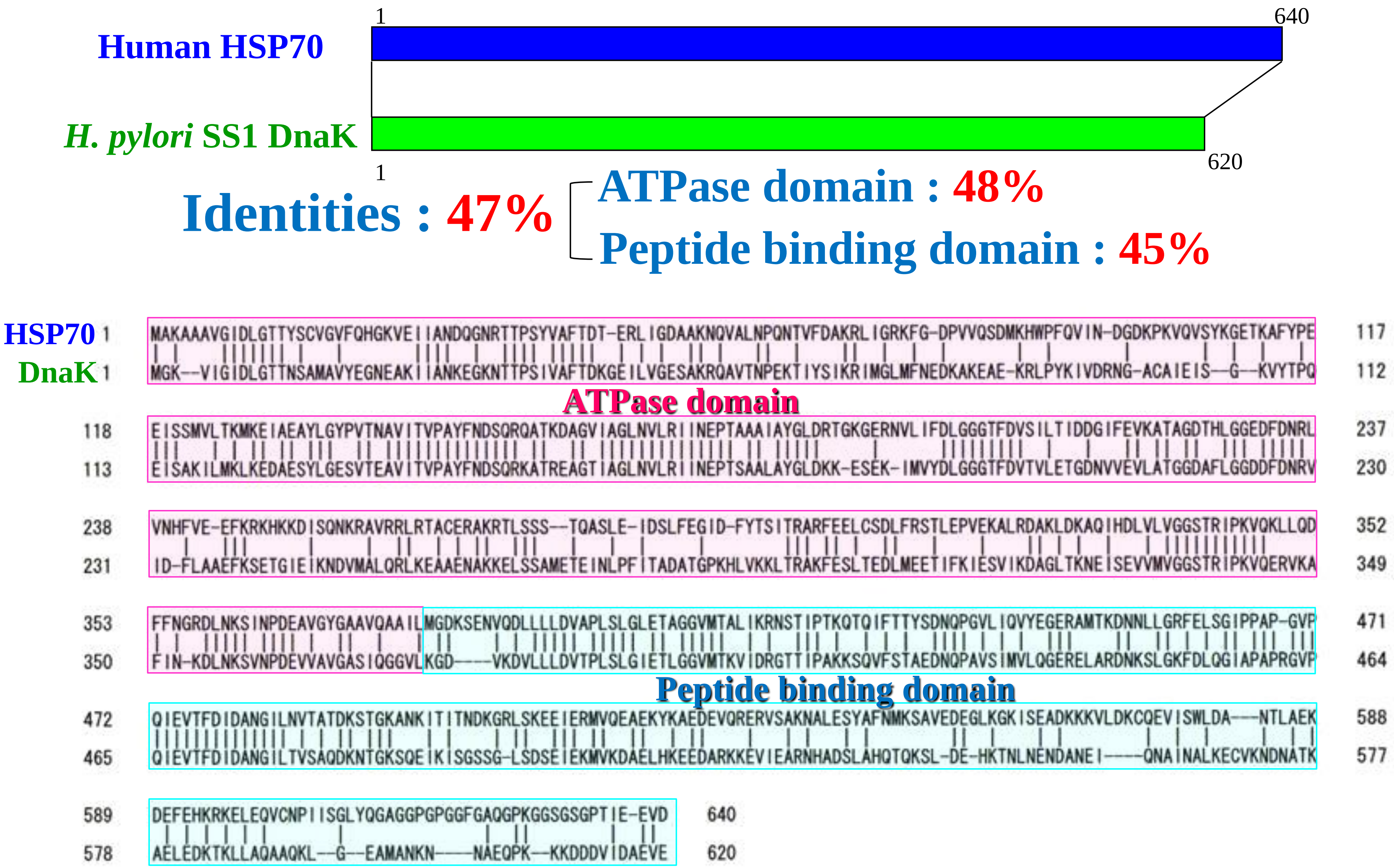

Supplement: Supplementary Information [file srep13738-s1.pdf]
